# Molecular Dynamics Simulation of the Mechanical Properties of Nanolayered Zr-Nb Alloys: Effects of Orientation and Layer Thickness

**DOI:** 10.3390/ma19071398

**Published:** 2026-03-31

**Authors:** Fugen Deng, Guiyu Liu, Jianhao Yan, Yulu Zhou, Yifang Ouyang

**Affiliations:** State Key Laboratory of Featured Metal Materials and Life-Cycle Safety for Composite Structures, Guangxi Key Laboratory for Relativistic Astrophysics, School of Physical Science and Technology, Guangxi University, Nanning 530004, China

**Keywords:** Zr–Nb alloys, strength–ductility synergy, BCC layer thickness, nucleation, critical resolved shear stress, dual-phase, peak stress, molecular dynamics, stacking faults

## Abstract

The mechanical performance of Zr–Nb dual-phase alloys is strongly influenced by the metastable β (body-centered cubic, BCC) phase and its crystallographic orientation, yet the underlying deformation mechanisms remain unclear. In this work, molecular dynamics (MD) simulations were conducted to investigate the compressive behavior of nanolayered Zr–Nb alloys with varying loading directions and BCC layer thickness (*T_BCC_*). The results reveal that interfacial coordinated strain governs the activation of various deformation modes. When the loading conditions promote strain compatibility at the interface between the hexagonal close-packed (HCP) and BCC phases, significant plasticity in the BCC phase assists the nucleation of stacking faults (SFs) and the activation of high critical resolved shear stress (CRSS) <c + a> slip systems in the HCP phase, leading to enhanced strength–ductility synergy of the material. In addition, *T_BCC_* induces a non-monotonic peak stress response, with a transition thickness of ~10.96 nm. Below this threshold, stress-induced phase transformation in the BCC phase is the dominant mechanism for strengthening. Above this thickness, increased interlayer spacing enhances dislocation interactions and spatial effects, resulting in improved strain hardening and plastic stability. These findings clarify the competition between transformation-induced and dislocation-mediated strengthening and provide atomic-scale guidance for the microstructural design of high-performance Zr–Nb alloys.

## 1. Introduction

Zirconium (Zr) alloys play a crucial role in the nuclear industry due to their low thermal neutron absorption cross-section, excellent corrosion resistance, and favorable mechanical properties [1,2,3]. Among these alloys, the Zr–2.5Nb alloy has been widely used in reactor pressure tubes and has undergone extensive experimental investigation [4]. However, the challenging service environment leads to material degradation, which has spurred the development of Zr–Nb alloys with enhanced mechanical performance and improved damage tolerance. Previous studies on this alloy system have explored various aspects, including phase transformation behavior and elemental distribution [5], the influence of microstructure on mechanical properties [6,7,8], and deformation anisotropy [9].

Recent experimental studies have demonstrated that Zr–2.5Nb alloys with hierarchical nanolamellar α/β (hexagonal close-packed, HCP/body-centered cubic, BCC) microstructures can achieve an exceptional balance of high fracture resistance and strength-ductility synergy. This improvement is primarily attributed to the activation of <c + a> dislocations at the α/β phase interface, which serve as effective dislocation sources at room temperature [10,11,12]. Although it is well established that the activation of <c + a> dislocations is highly dependent on the loading direction, and that the overall strength of the alloy is primarily determined by the α phase [13], the fundamental mechanism that triggers <c + a> slip in nanolayered Zr–2.5Nb alloys remains unclear.

At the nanoscale, the mechanical behavior of layered materials is significantly influenced by their geometric dimensions. Studies on nanoscale layered systems, such as Zr/Nb [14] and Mg/Nb [15], showed that strength is maximized at specific layer thicknesses, commonly attributed to interface-induced impediments to dislocation motion. Moreover, recent experimental and molecular dynamics (MD) studies on FCC/BCC nanolayered eutectic high-entropy alloys demonstrated that stable plastic flow in the BCC phase, its coordinated interaction with deformation mechanisms in adjacent phases, and the interphase spacing between constituent phases, plays a critical role in determining the overall mechanical response of these materials [16]. These findings indicate that deformation of the secondary phase in dual-phase alloys significantly influences the plastic behavior of the matrix phase. Experimental studies on Zr–2.5Nb confirmed that the evolution of interphase stresses during room-temperature deformation is strongly affected by phase characteristics and loading orientation [17], highlighting the vital role of the β phase in regulating plastic deformation within the α phase. Although the β phase occupies a relatively small volume fraction at room temperature, its effect on mechanical response becomes more pronounced at the nanoscale. Therefore, systematic investigations on the dependence of mechanical behavior on loading-direction and the role of the β phase in Zr–Nb alloys are necessary.

MD simulations have become an indispensable tool for investigating atomic-scale deformation mechanisms in nanoscale metallic materials. These simulations offer insights into microscopic details that are often challenging to obtain through experimental methods. MD simulations are now widely applied to study the mechanical behavior of metallic multilayers and dual-phase alloys, particularly those with complex interfaces. MD simulations have shown that under tensile [18,19,20] and shear loading [21,22], different types of interfaces exhibit distinct mechanisms of dislocation–interface interactions, thereby influencing the macroscopic mechanical properties. In HCP/BCC systems, variations in the orientation relationship can regulate the nucleation behavior of interfacial dislocations. For example, at the Zr/Nb interface, the Pitsch-Schrader and Burgers orientations activated <c + a> dislocations of the same type but along different slip directions during axial deformation [23]. Nanotribological scratching simulations on Zr/Nb multilayer films demonstrated that the interface with the Rong-Dunlop orientation shows superior wear resistance [24]. In addition to interfacial structure, the loading conditions significantly affect deformation behavior. Tensile simulations of dual-phase systems such as TiAl/Ti_3_Al [19] and Cu/W [20] along three crystallographic orientations revealed a pronounced orientation dependence of strength. This behavior originates from the migration of dislocation nucleation sites. In the Zr/Nb system, for example, changing the loading direction reconstructed the distribution of atomic strain at the interface, which in turn altered the preferred sites for dislocation nucleation [23]. In addition, layered architectures impose geometric constraints on dislocation motion. In FCC/BCC layered high-entropy alloys, MD simulations showed that as the BCC layer thickens, the transition of the BCC phase to the HCP phase is suppressed and the dislocation density is changed, ultimately leading to an increase in strength [25]. Studies on AlCoCrFeNi_2.1_ revealed that increasing the BCC layer thickness can trigger a transition in the strengthening mechanism from Hall–Petch strengthening to inverse Hall–Petch softening [16].

In this study, MD simulations have been employed to investigate the mechanical behavior and deformation mechanisms of nanolayered Zr–Nb dual-phase alloys. The focus is on the impact of loading direction and the thickness of BCC phase layers. By analyzing atomic-scale processes such as interfacial strain coordination, dislocation evolution, and phase transformation, this study elucidated the key factors governing the synergy between strength and ductility, as well as the mechanism behind the non-monotonic dependence of the mechanical response on BCC layer thickness. These findings provide atomic-scale insight into the deformation mechanisms of dual-phase Zr alloys and offer a theoretical basis for optimizing their mechanical performance through microstructural design.

## 2. Methodology

**Figure 1:**
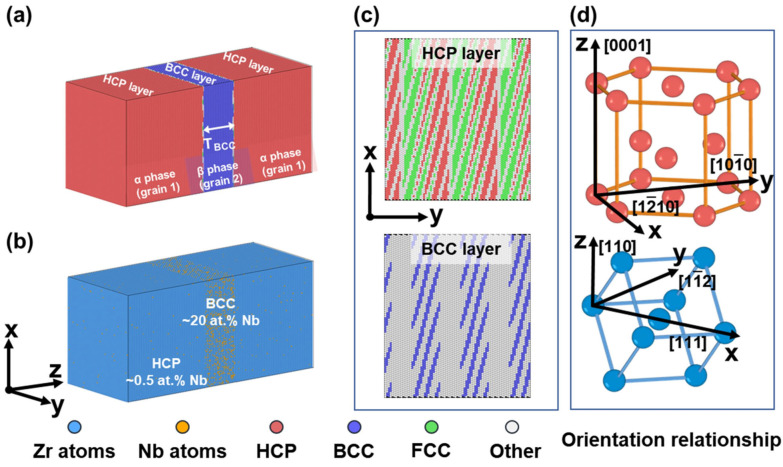
(**a**) Layered model of Zr–Nb alloy with grains colored according to phase structure; (**b**) Distribution of Nb atoms in both phases; (**c**) Interface structure between adjacent HCP and BCC atomic layers at the phase boundary, colored according to CAN; (**d**) Orientation relationship between the dual-phase structures.

All MD simulations were conducted using the open-source software LAMMPS (version 29 August 2024) [26]. The interaction potential function describing Zr and Nb atoms was the angular-dependent potential (ADP) developed by Starikov et al. [27], which has been validated for the mixed enthalpy of the BCC Zr–xNb. This potential has been applied in previous MD studies of the Zr–Nb system to simulate the mechanical behavior of dislocations, second phases, and amorphous states [28,29], as well as the composition of Zr–Nb phases within the BCC structure [30] and the friction mechanics at the Zr/Nb interface [24]. In the MD simulations examining interfacial mechanical properties, a layered model is a commonly used approach [31,32,33,34,35,36]. This study employed a layered interface model to analyze the evolution of the intrinsic structures in dual-phase systems and their mechanical behavior. The atomic structures of the two phases are shown in Figure 1a. Drawing from experimental investigations of the Zr–Nb alloy interface [11,14] and atomic simulations of the HCP/BCC interface [23], the classical Burgers orientation relationship (BOR) was adopted for the HCP/BCC dual-phase model. The specific lattice orientation is shown in Figure 1d, where the x-direction $\left[1\overset{-}{2}10\right]$_HCP_ is parallel to $[\overset{-}{1}11]$_BCC_, and the HCP plane (0001) is parallel to the BCC plane (110). In this model, the thickness of the intermediate BCC phase layer is designated as *T_BCC_*. To investigate its influence on the mechanical properties of the material, seven distinct *T_BCC_* values were selected: 3.99, 4.98, 5.98, 7.48, 8.97, 10.96, and 13.46 nm, while maintaining a constant total thickness of the two phases along the z-axis. It should be pointed out that, when a constant total layer thickness is maintained, the change in *T_BCC_* will inevitably lead to changes in the volume fractions of both HCP and BCC phase, although such methods are often used in calculations [37,38]. This study focuses on the dominant role of spatial effects due to layer thickness in deformation behavior, and considering the computational efficiency, this approach was adopted. Figure 1c depicts the HCP/BCC interface, coloured according to atomic structure. In the y-direction, HCP and BCC layers exhibit a periodic structural distribution. The “other” structure within the HCP layers (white in Figure 1c) represents a metastable interface configuration characterized by a high stacking-fault energy (SFE) [39]. The Nb concentrations in the BCC and HCP phases were approximately 20 at.% and 0.5 at.%, respectively, as depicted in Figure 1b. This composition matches the Nb content observed in both phases of the Zr–2.5Nb alloy at room temperature [5,10,11,40].

Research on high-temperature phase transformations in multi-element alloys indicates that elements transition from a disordered to an ordered state during low-to-high-temperature processes, primarily driven by strong bond energies in binary alloys [41]. The metastable β phase in low-Nb Zr alloys is a BCC Zr–Nb solid solution [40]. However, at room temperature, the β phase with low concentrations of Nb is unstable, and only strong Zr–Nb bonding can stabilize the BCC structure [42]. In this study, a hybrid Monte Carlo/Molecular Dynamics (MC/MD) approach [43] was employed to achieve an ordered and relatively stable metastable β phase at high temperatures. As shown in Figure 1b, Zr atoms in the HCP layer were randomly substituted by Nb atoms at a concentration of 0.5 at.%, while in the BCC layer, Zr atoms were substituted with Nb atoms at a concentration of 20 at.%. The conjugate gradient method was then used for energy minimization. After a relaxation period of 10 ps at 1223 K, a preliminary stable model was established. Following this, while maintaining a constant temperature, hybrid MC/MD operations were conducted to develop a reasonable HCP/BCC biphasic structural model under these thermal conditions [10]. The atoms were divided into HCP and BCC groups. Within each group, MC-swap operations were performed, which involved randomly selecting two atoms of different chemical compositions for position swapping. The success of each swap was evaluated using the Metropolis criterion [44]. After every 100 MC swap attempts, MD relaxation was performed for 500 time steps to eliminate local residual stresses. This MC/MD procedure was repeated until the total number of MC swap attempts reached 200,000. Following this process, the atomic potential energy decreased from approximately −4.3146 eV/atom to −4.3167 eV/atom. The model was then cooled to 300 K over 20 ps, followed by an additional 20 ps of thermostatted relaxation at 300 K under the isothermal-isobaric (NPT) ensemble. The evolution of energy (both potential and total), temperature, and average atomic volume during cooling and relaxation is presented in Figure S2 of the Supplementary Materials. All quantities remain stable and demonstrate clear convergence during the final 20 ps at 300 K, confirming that the system reaches a well-equilibrated state prior to mechanical loading. The final configuration served as the initial structure for simulating mechanical behavior. Additionally, to eliminate stochastic effects introduced by the MC/MD procedure, two sets of HCP/BCC structures, generated from MC/MD simulations of different durations, were subjected to the same compressive loading conditions. As shown in Supplementary Figure S1, the peak stress and flow stress showed consistent trends in both cases, confirming the reliability of the model.

All models have dimensions of approximately 26 nm × 23.3 nm × 55 nm, with the strain mismatch parameter calculated according to the formula 2|*L_HCP__–i_* − *L_BCC__–i_* |/|*L_HCP__–i_* +*L_BCC__–i_*| (where *i* denotes the x or y direction) [23]. Here, *L_HCP__–i_* represents the length of the HCP grain along the x or y direction, and *L_BCC__–i_* denotes the length of the BCC grain along the x or y direction. The calculated misfit strains in the x and y directions are 0.050% and 0.012%, respectively. To elucidate the anisotropy of interfacial deformation mechanisms, uniaxial compression loading was applied with a 40% engineering strain along three orthogonal directions: x, y, and z. A time step of 1 fs was used, and periodic boundary conditions were employed in all three directions. Compression was performed under NPT ensemble conditions at a constant strain rate of 2 × 10^9^ s^−1^. The other two non-deformed axes were kept at zero stress. Post-processing visualisation was performed using the Open Visualization Tool (OVITO, version 3.12) [45]. The common neighbor analysis (CNA) algorithm [46] was utilized to distinguish crystal structures. The dislocation extraction algorithm (DXA) [47] was used to identify and quantify the types, densities, and Burgers vectors of dislocations. The von Mises strain was utilized to evaluate local strain levels at the atomic scale, calculated as follows [48]:

(1) $$\eta^{\mathrm{Mises}}={\left[\eta_{\mathrm{xy}}^{2}+{\;\eta }_{\mathrm{xz}}^{2}+{\;\eta }_{\mathrm{yz}}^{2}+\;\frac{1}{6}\left({\left(\eta_{\mathrm{xx}}-\eta_{\mathrm{yy}}\right)}^{2}+{\left(\eta_{\mathrm{xx}}-\eta_{\mathrm{zz}}\right)}^{2}+{\left(\eta_{\mathrm{yy}}-\eta_{\mathrm{zz}}\right)}^{2}\right)\right]}^{1/2}$$

where *η* represents the Green-Lagrange strain tensor.

## 3. Results and Discussion

### 3.1. Effect of Loading Direction

#### 3.1.1. Stress–Strain Curve

**Figure 2:**
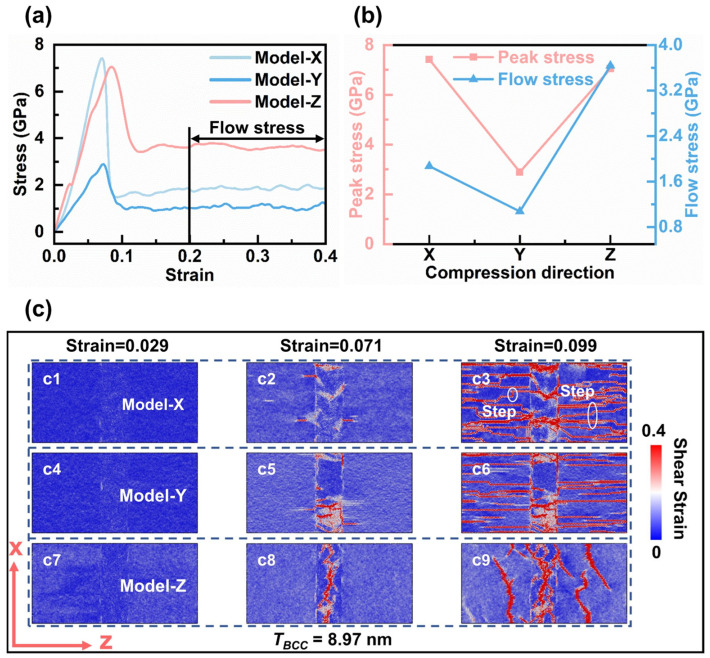
Uniaxial compressive mechanical responses of the three models. (**a**) Stress–strain curves. (**b**) Peak stress and flow stress. (**c**) Atomic shear strain snapshots of the models with a *T_BCC_* thickness of 8.97 nm at different strain levels. Panels (**c1**–**c3**) correspond to Model X, (**c4**–**c6**) to Model Y, and (**c7**–**c9**) to Model Z. Atoms are colored according to their atomic shear strain values.

The deformation behavior of a nanolayered Zr–Nb biphasic model with *T_BCC_* = 8.97 nm is investigated under uniaxial compressive loading in the -x, -y, and -z directions. The stress–strain curves are presented in Figure 2a. The results indicate that Model X exhibits the highest peak stress at 7.41 GPa, while Model Y shows the lowest peak stress at 2.89 GPa. Model Z has a peak stress slightly lower than Model X, at 7.05 GPa. Figure 2b compares the peak stress and flow stress across the three models. The flow stress, calculated from the average stress within the strain range of 0.2 to 0.4, is closely related to the dislocation density and stacking configuration [49,50,51]. Model Z demonstrates the highest strain at peak stress, indicating superior plasticity and a strong synergy between strength and ductility. This strength–ductility synergistic behavior is consistent with the macroscopic observations of the Zr–Nb alloy experiments [10,11]. In addition, the high compressive strength of Model Z aligns with experimental findings, which show that maximum strength is achieved when compressive or tensile loading is applied along the <c> axis of α-Zr [17]. Overall, our results demonstrate that the strength–ductility synergy is strongly correlated with the direction of deformation in Zr–Nb dual-phase alloys.

#### 3.1.2. Local Deformation Modes

To investigate the different deformation mechanisms across the three models, local shear strain contour plots are generated at various strain stages to reveal the localisation of plastic deformation within the material (Figure 2c). As demonstrated in Figure 2(c2,c5,c8), both the HCP and BCC phases exhibit pronounced strain non-uniformity during deformation, with the HCP phase displaying hysteretic deformation behavior. This behavior is primarily due to the BCC structure having multiple slip systems available at room temperature, whereas the HCP structure has a limited number of activatable slip systems. Consequently, the BCC phase in the Zr–Nb two-phase system tends to undergo plastic deformation first. As illustrated in Figure 2(c3,c6), there are notable differences between the plastic deformation of the BCC and HCP phases in Models X and Y. Specifically, the localized strain distributions differ between the two phases, creating a distinct strain boundary forming at the phase interface. In contrast, as shown in Figure 2(c9), the two phases in Model Z exhibit similar deformation modes, with no pronounced strain partitioning observed at the phase interface.

In Model X, no significant localised strain was observed in either phase during the initial deformation stage (Figure 2(c1)). As strain increases, local deformation first occurs within the BCC region and then propagates across the phase boundary into the HCP region (Figure 2(c2)). In the later stages of deformation, strain localisation within the BCC region became more pronounced (Figure 2(c3)). Step-like strain localisation emerged within the HCP region (white-circled areas in Figure 2(c3)), arising from the interaction of the dislocations on slip planes and the propagation of intergranular stacking faults (SFs). When comparing the strain localisation diagrams for Model Y, shown in Figure 2(c6), the step-like localisation is less pronounced than in Model X. Although both exhibit consistent dislocation evolution directions within the HCP region, the differing numbers of “steps” indicate that the -x and -y load directions induce two distinct deformation patterns within the HCP phase. Notably, when the strain reaches 0.071, the dominant deformation in Model Z remains concentrated within the BCC phase region (Figure 2(c8)). In contrast, in Model X and Model Y, deformation has already spread across the HCP/BCC interface, propagating from the BCC phase into the HCP phase (Figure 2(c2,c5)). At this point, both Model X and Model Y are in the stress reduction stage, while Model Z is still experiencing deformation before reaching peak stress. This observation suggests that peak stress may primarily originate from deformation within the BCC phase, where stress accumulates to a critical level before dissipating into the HCP phase. In compression models of layered composite materials, the first stress peak is typically associated with interfacial dislocations, whereas the second peak corresponds to the nucleation of dislocations near the interface and their subsequent propagation into the softer layer [52]. Furthermore, the deformation of the HCP phase is triggered by the deformation of the BCC phase, indicating that the microdeformation patterns in the BCC phase influence the subsequent plastic deformation behavior of the HCP phase (Figure 2(c2,c5,c8)).

**Figure 3:**
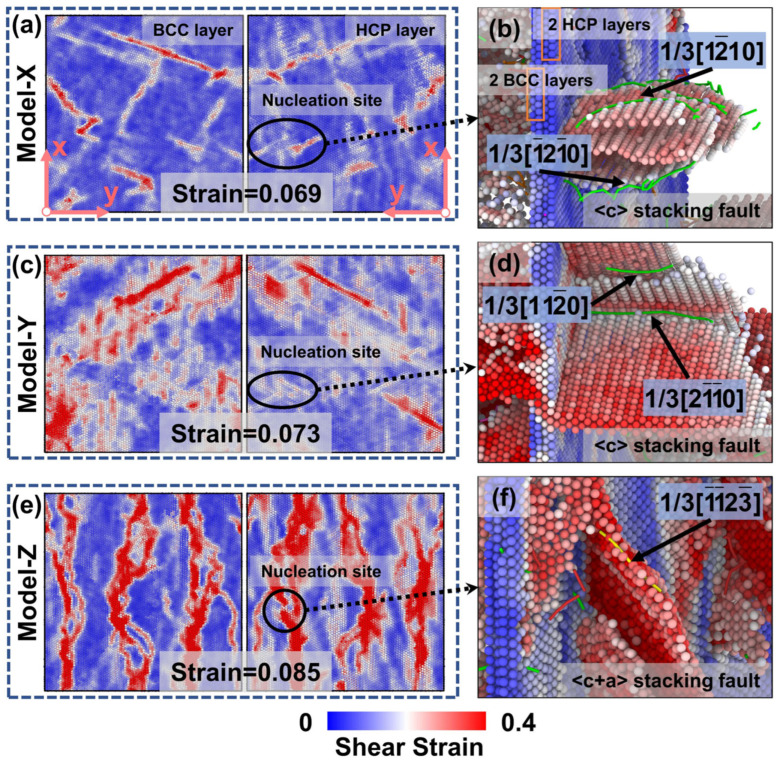
Comparison of interfacial strain and HCP deformation mechanisms at peak compressive stress. (**a**,**b**) Model X, (**c**,**d**) Model Y, and (**e**,**f**) Model Z. The left panels (**a**,**c**,**e**) show local strain maps of adjacent BCC layers (**left**) and HCP layers (**right**) at the interface, while the right panels (**b**,**d**,**f**) show the SFs and dislocation structures on the HCP side.

To elucidate the factors influencing the plastic deformation of the HCP phase, a microscopic analysis is conducted by examining the local interfacial structures near the peak stress for each model, as shown in Figure 3. Figure 3a presents the local interfacial strain distribution in Model X at a strain of 0.069. The SFs and dislocations in the HCP phase, depicted in Figure 3b, originate from the black-circled region in Figure 3a. In this region, atoms with a shear strain greater than 0.25, referred to as S atoms, are identified as those associated with SFs. Similarly, Figure 3c–d and Figure 3e–f correspond to Models Y and Z, respectively. SFs preferentially nucleate in the regions of high local strain, with nucleation sites typically found in disordered structural areas along the phase interface [53]. These regions are high-energy zones at the interface [39], making them inherently unstable and more prone to deformation. As illustrated in Figure 3a,c,e, the localized high-strain regions in the corresponding atomic layers of the BCC and HCP phases exhibit clear spatial correspondence across all three models. However, the strain intensity in the red high-strain areas of the BCC and HCP layers shows notable asymmetry in Models X and Y, whereas a symmetric strain distribution is observed in Model Z. This symmetry, or asymmetry, in interfacial strain indicates whether plastic deformation is accommodated cooperatively between the BCC and HCP phases at the phase interface. Accordingly, these two modes of deformation are referred to as interfacial coordinated strain and interfacial uncoordinated strain, respectively. This difference can be traced back to the influence of loading direction on the nucleation capability of disordered interface regions, which results in changes to the interfacial structures among the three models. In realistic materials, the interface may also exhibit a stepped shape [11,53], which can further exacerbate the asymmetry of interface strain and complicate the deformation mode.

When subjected to compressive loading, the direction of the load influences the level of deformation in the interfacial atoms, which subsequently affects the types of lattice dislocations that are emitted from the interface into the grain interior [23]. As illustrated in Figure 3b,d, the slip behavior of the SFs and the resulting dislocations in Model X and -Y are similar. The SFs nucleate at the phase boundary and propagate along the <c> axis of the HCP structure within the grain, accompanied by 1/3<$1\overset{-}{2}10$> partial dislocations. In contrast, the SF in Model Z slips along the <c + a> direction, resulting in the production of 1/3<$1\overset{-}{2}13$> dislocations. In HCP crystals, pyramidal <c + a> (Py I and Py II) slip systems have a significantly higher critical resolved shear stress (CRSS) for activation than the prismatic <a> (P) slip system [53,54,55]. Model X and -Y exhibit lower local deformation in the BCC phase, resulting in interfacial uncoordinated strain between the two phases. The relatively low interfacial strain on the HCP side facilitates the nucleation and slip of the low-CRSS P slip into the HCP interior. In contrast, as shown in Figure 3f, the interfacial coordinated strain in Model Z induces the nucleation of high-CRSS Py II slip at the phase boundary, followed by slip towards the HCP interior. This behavior is consistent with the interfacial dislocation formation observed in studies of the Zr/Nb interface [23].

However, attributing the high peak stress solely to the activation of <c + a> dislocations in the HCP phase is insufficient; it is also important to consider the plastic deformation capacity of the BCC phase. For the BCC phase, the maximum Schmid factors for the primary slip systems under uniaxial compression along the X, Y, and Z directions are 0.314, 0.393, and 0.471, respectively [23]. A larger Schmid factor indicates that the slip system is easier to activate. In Model X, the deformation of the BCC phase shows a smaller Schmid factor, suggesting lower plastic deformability and a greater requirement for axial stress to activate its slip systems. Model Z has higher Schmid factors in its BCC phase, allowing for plastic deformation at lower stresses; however, it also necessitates higher axial stresses to activate the <c + a> slip system in the HCP phase. As a result, both Model X and Model Z demonstrate elevated peak stress levels. Moreover, for Model X and Model Y, which display an interfacial uncoordinated strain mode, the lower Schmid factor of Model X explains why it requires higher axial stress to activate the slip system in the BCC phase, resulting in a peak stress that is higher than that of Model Y. Overall, achieving a strong strength–ductility synergy in a two-phase system requires meeting two conditions: pronounced plastic deformation within the BCC phase and interfacial coordinated strain between phases.

#### 3.1.3. Dislocation Analysis

**Figure 4:**
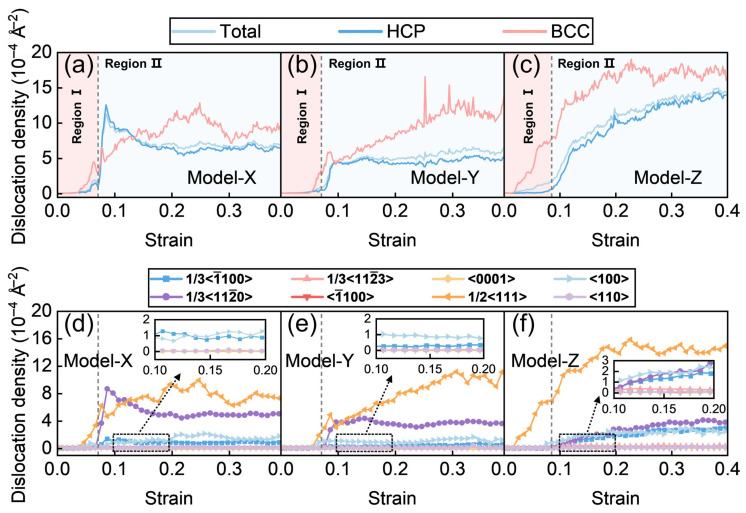
Evolution of dislocation density during deformation across different models. (**a**–**c**) Total dislocation density and the dislocation densities of the two phases for Models X, Y, and Z, respectively. (**d**–**f**) Dislocation density curves for different dislocation types in Models X, Y, and Z, respectively (the regions partitioned by dashed lines are consistent with those in **a**–**c**).

To investigate the deformation mechanisms in depth, a quantitative analysis of dislocation density evolution with strain is performed. Figure 4a–c presents the strain-dependent dislocation density curves for Models X, Y, and Z, respectively. The total dislocation density is calculated by dividing the combined dislocation length in the HCP and BCC phases by the total model volume. The HCP and BCC dislocation densities were obtained by dividing the dislocation line length in each phase by its initial volume. Figure 4d–f shows the dislocation densities of different dislocation types for the three models. In these figures, Burgers vectors with three-index notation originate from the BCC phase, whereas those with four-index notation originate from the HCP phase.

By correlating the mechanical response with the evolution of dislocation density, as illustrated in Figure 4a–c, the deformation process is divided into two stages: region I (before peak stress) and region II (after peak stress), separated by the point of peak stress onset. In region I, Model Z consistently exhibits a higher total dislocation density than Model X and Model Y, which is predominantly governed by BCC phase dislocations and evolves with a gentle slope. This stage is characterized by the extensive activation of 1/2<111> dislocations in the BCC phase (Figure 4f), reflecting a strong capability for intrinsic plastic deformation. The observed strengthening behavior during this gentle slope stems from abundant slip reactions along SFs within the BCC phase, which partially suppress rapid dislocation proliferation. Once the peak stress onset point is surpassed, Model X and Model Y exhibit a pronounced decrease in dislocation density during the stress drop stage. This decline is primarily due to the annihilation of BCC 1/2<111> dislocations near the phase interface when plastic deformation in the HCP phase is triggered by interfacial activation (Figure 4d–f). In contrast, Model Z shows similar slip directions in both phases, allowing dislocation transmission across the interface with minimal impedance. As a result, its dislocation density continues to increase. In region II (the flow-stress stage), the evolution of the total dislocation density is primarily governed by dislocation activity in the HCP phase. The ranking of total dislocation density (Model Z > Model X > Model Y) aligns with the sequence of flow-stress levels, demonstrating that dislocation density is the key microstructural parameter governing work-hardening capacity. Further analysis of dislocation density evolution for different dislocation types, as shown in Figure 4d–f, reveals that the dominant dislocation type in the HCP phase of Model X and Model Y is the 1/3<$11\overset{-}{2}0$> partial dislocation associated with P slip. In contrast, the magnified views in Figure 4d–f show that Model Z exhibits not only prismatic dislocations but also 1/3<$11\overset{-}{2}3$> dislocations linked to <c + a> SF slip, reflecting a more complex dislocation evolution.

**Figure 5:**
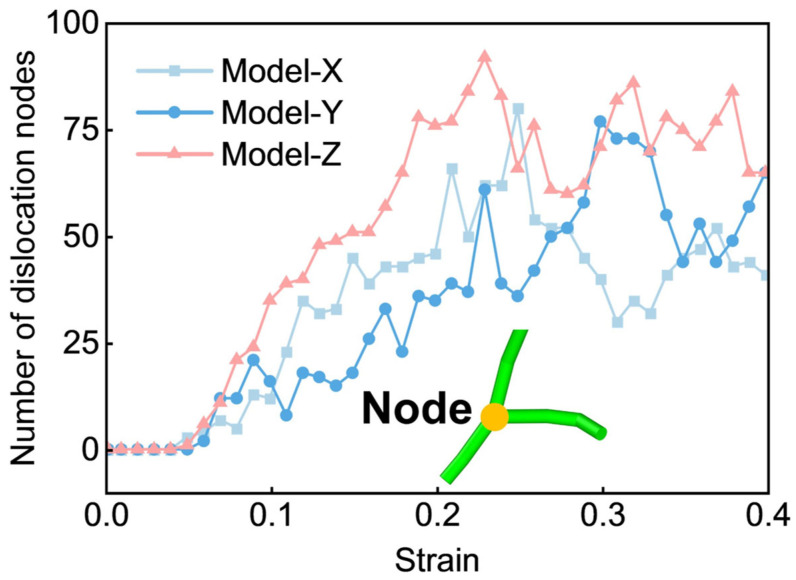
Evolution of the number of dislocation nodes with strain for models deformed along different loading directions.

A comparative analysis of the 1/3<$11\overset{-}{2}0$> partial dislocation densities during the flow-stress stage across the three models shows that while Model Z exhibits a greater variety of dislocation types, its density of 1/3<$11\overset{-}{2}0$> partial dislocations remains lower than that of Model X and Model Y. To further explain this phenomenon, a dislocation node (a node formed by two or more dislocation heads or tails) is introduced for analysis. The formation of dislocation nodes reflects the material’s hardening behavior [56]. An increase in the number of nodes indicates abundant dislocation reactions, resulting in complex entangled dislocation structures. As shown in Figure 5, Model Z exhibits a continuous increase in the number of dislocation nodes throughout deformation, reaching significantly higher levels than Model X and Model Y during the flow-stress stage. This behavior originates from intensive dislocation reactions involving 1/3<$11\overset{-}{2}0$> partial dislocations, which promote the generation of multiple secondary dislocation types while reducing the population of 1/3<$11\overset{-}{2}0$> dislocations. The resulting dislocation entanglement enhances node formation and generates a stronger work-hardening effect. This microstructural evolution is the fundamental origin of the elevated flow-stress observed in Model Z.

#### 3.1.4. Relationship Between Stress Distribution and Dislocation Evolution

**Figure 6:**
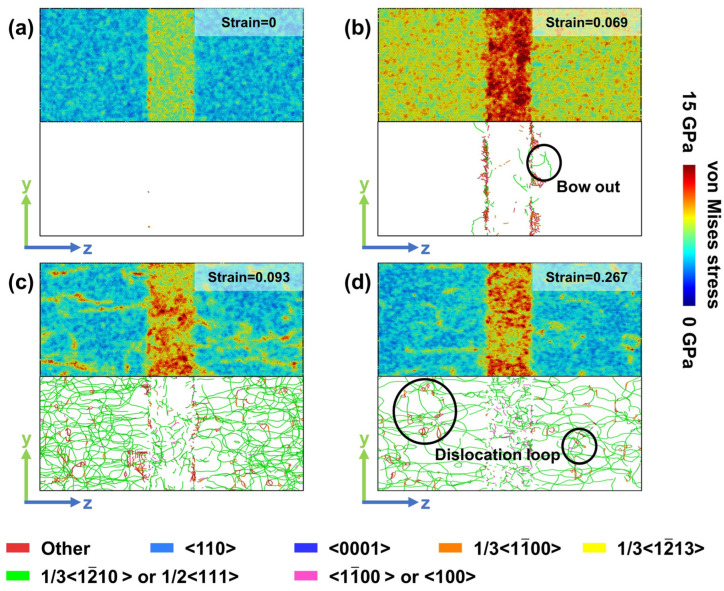
Local stress and dislocation distributions in Model X at different strain levels. (**a**) Strain = 0. (**b**) Strain = 0.069. The circled region near the interface shows the formation of a dislocation bow. (**c**) Strain = 0.093. (**d**) Strain = 0.267. Dislocation loops form in the circled region within the HCP phase.

**Figure 7:**
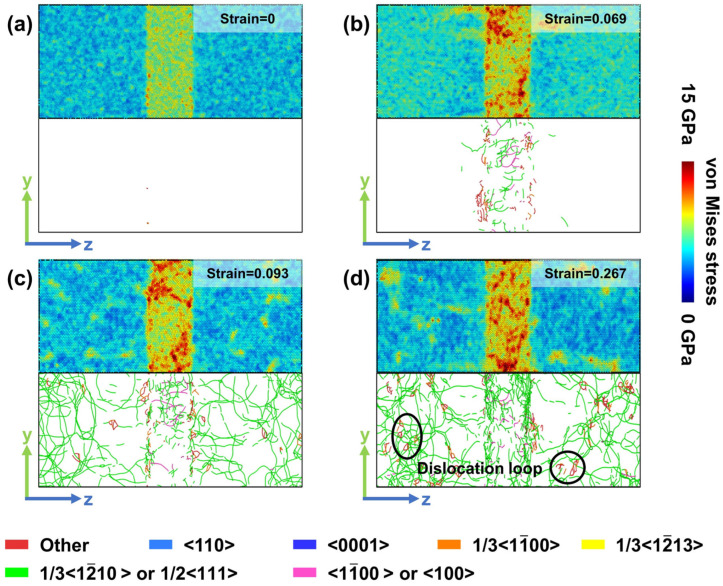
Local stress and dislocation distributions in Model Y at different strain levels. (**a**) Strain = 0. (**b**) Strain = 0.069. (**c**) Strain = 0.093. (**d**) Strain = 0.267. Dislocation loops form in the circled region within the HCP phase.

**Figure 8:**
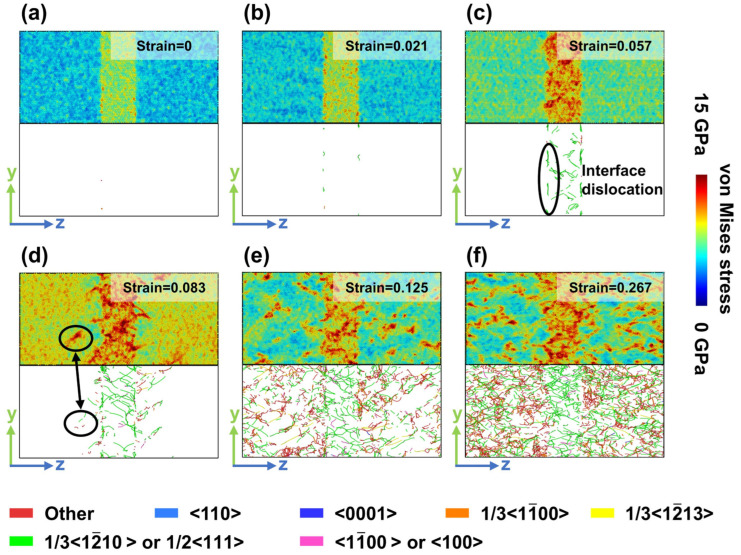
Local stress and dislocation distributions in Model Z at different strain levels. (**a**) Strain = 0. (**b**) Strain = 0.021. (**c**) Strain = 0.057, with the circled region indicating an interfacial dislocation. (**d**) Strain = 0.083, with the two circled regions in the HCP phase denoting the same area, which acts simultaneously as the origin of dislocation lines and a site of stress concentration. (**e**) Strain = 0.125. (**f**) Strain = 0.267.

Figure 6, Figure 7 and Figure 8 illustrate the correlation between stress distribution and dislocation evolution during deformation for Model X, Model Y, and Model Z, respectively. In Figure 6 and Figure 7, subfigures a–d correspond sequentially to four characteristic stages of the stress–strain response: the undeformed state, peak stress, stress drop, and flow-stress. To analyze the microstructural evolution of Model Z at the yield point, Figure 8 presents the stress distribution and dislocation configurations associated with its two yield points (Figure 8b,c). Figure 8d–f correspond to the peak stress, stress drop, and flow-stress stages for this model, respectively.

In Model X, the comparison between Figure 6a,b shows that dislocations initially nucleate primarily at the two-phase interface during deformation up to the peak stress. At the peak stress stage (Figure 6b), stress accumulates mainly within the BCC phase, while dislocations bow outward from the phase boundary into the HCP phase. A similar dislocation bowing behavior has also been reported in experimental mechanical tests on Zr-–2.5Nb alloys [11]. As deformation progresses into the stress drop stage (Figure 6c), a pronounced dislocation burst occurs in Model X, characterized by the formation of numerous 1/3<$1\overset{-}{2}10$> partial dislocations within the HCP phase. Concurrently, the stress intensity within the BCC phase decreases markedly, although its internal dislocation density increases compared to the peak-stress state. When entering the flow-stress stage (Figure 6d), dislocation interactions lead to the formation of stable dislocation loop structures. In this stage, a spatial correlation is observed between localized stress concentration regions and areas of dense dislocation line distribution.

Model Y exhibits different deformation characteristics. As shown in Figure 7b, at a strain of 0.069, the stress concentration within the BCC phase is weaker than that in Model X, yet a larger number of dislocations are generated. In the stress drop stage (Figure 7c), the stress level in the BCC phase does not significantly decrease compared to Figure 7b, while the number of dislocations formed in the HCP phase remains lower than that observed in Model X at the same strain (0.093). In the subsequent flow-stress stage (Figure 7d), extensive dislocation reactions lead to the formation of a larger number of dislocation loop structures.

In Model Z, regularly arranged and intermittently distributed dislocation structures formed at the HCP/BCC interface (Figure 8c). The intersecting 1/3<$1\bar{2}10$> partial dislocations within the BCC phase in Figure 8c indicate the formation of cross-slip bands. At peak stress (Figure 8d), stress concentration within the BCC phase is situated between those of Models X and Y. Internally distributed dislocations continue to proliferate, while dislocations propagating along the <c + a> direction from interstitial positions at the interface extend into the HCP phase, indicating the activation of <c + a> SF within the HCP phase. At this stage, regions of stress concentration coincide spatially with areas of dislocation accumulation, as indicated by the circled areas connected by double arrows in Figure 8d. When entering the stress-drop stage (Figure 8e), the cross-slip of SF triggers additional dislocation multiplication within the HCP phase. This process continues into the flow-stress stage (Figure 8f), ultimately producing a large number of dislocation structures. A comparison of stress distributions at the identical strain of 0.267 between Model X (Figure 6d) and Model Y (Figure 7d) shows that Model Z (Figure 8f) exhibits significantly more pronounced localized stress concentrations within the HCP region.

The analysis indicates that during the initial strain stage, plastic deformation begins in the BCC phase. This phase’s ability to accommodate dislocation growth reflects the material’s overall initial capability for plastic deformation. Additionally, during early strain, the BCC phase bears a higher stress intensity than the HCP phase. These findings align with experimental results regarding the mechanical deformation behavior of the Zr–2.5Nb alloy at room temperature [17]. The observation of a high dislocation density in the BCC phase helps explain the strong plasticity exhibited in Model Z at peak stress. A cross-distributed dislocation network forms within the BCC phase, which simultaneously enhances the material’s plasticity and mitigates stress concentration within that phase itself. Furthermore, regular deformation at the interface facilitates the activation of <c + a> SF slip within the HCP phase. The cross-slip behavior of the <c + a> SF contributes to the multiplication of dislocations during the flow-stress stage. In contrast, the high density of dislocations and the formation of numerous dislocation nodes collectively lead to an increase in flow stress. Therefore, the stage-by-stage results indicate that, in a two-phase structure, interfacial coordinated strain serves as a microscopic criterion for assessing the coordinated deformation capabilities of both phases. Moreover, the plastic behavior of the BCC phase plays a crucial role in inducing <c + a> slip in the HCP phase, which is a fundamental mechanism for the synergistic enhancement of both strength and plasticity, as proposed by Model Z.

### 3.2. Influence of BCC Layer Thickness

#### 3.2.1. Mechanical Response and Peak Stress Transition

**Figure 9:**
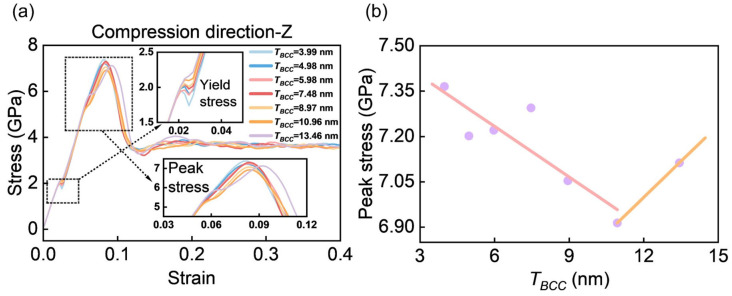
(**a**) Stress–strain curves for different *T_BCC_* compressions along the z-axis, with two insets highlighting yield stress and peak stress, respectively. (**b**) Peak stress values corresponding to different *T_BCC_* from (**a**), where the data points represent peak stress and the two straight lines are fitted based on the first six and last two points, respectively.

To clarify the role of the BCC phase in the Zr–Nb dual-phase system, the effect of *T_BCC_* on the compressive mechanical response under z-axis loading is investigated. Analysis of the compressive stress–strain curves (Figure 9a) and the corresponding peak stresses (Figure 9b) reveals a non-monotonic dependence of peak stress on *T_BCC_*. While all models exhibit similar overall stress–strain profiles, softening behavior is observed for *T_BCC_* < 10.96 nm, whereas a strengthening trend emerges for *T_BCC_* > 10.96 nm. This non-monotonic response is consistent with previous observations in CoCrFeNi/Al multilayer studies, where peak stress also exhibits an inflectional dependence on layer thickness [31].

The enlarged view in Figure 9a indicates that the model with *T_BCC_* = 13.46 nm exhibits a higher yield stress before reaching peak stress compared to models with other *T_BCC_* values. This yield strengthening delays the onset of peak stress, allowing it to occur at a higher strain, which indicates enhanced plasticity. Previous experimental studies on two-phase eutectic materials showed that reducing interlayer spacing enhances material strength [16]. Indeed, the spatial effects on the mechanical behavior of layered materials are associated with microstructural factors, such as dislocation activity [57] and phase transformations [25]. Therefore, the following sections will concentrate on these microstructures to elucidate the mechanism behind the transition in peak stress observed at *T_BCC_* = 10.96 nm**.**

#### 3.2.2. Dislocation and Phase Transformation Analysis

**Figure 10:**
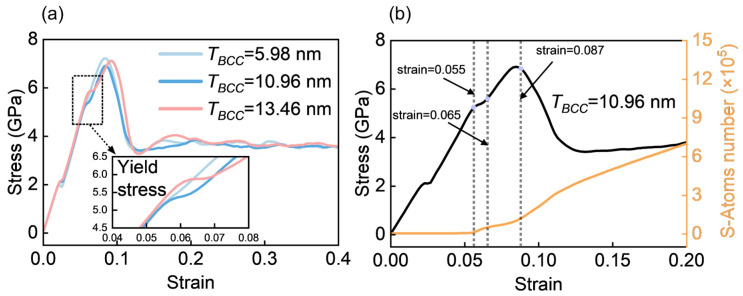
(**a**) Stress–strain curves for three representative models with *T_BCC_* values of 5.98 nm, 10.96 nm, and 13.46 nm. (**b**) Comparison of the stress–strain curve with the evolution of the number of SF atoms for the model with *T_BCC_* = 10.96 nm.

Three representative models with *T_BCC_* values of 5.98 nm, 10.96 nm, and 13.46 nm are systematically analyzed, representing the softening regime, the transition thickness, and the strengthening regime, respectively. The corresponding stress–strain responses are shown in Figure 10a, while Figure 10b correlates the stress–strain curve of the *T_BCC_* = 10.96 nm model with the evolution of S atoms.

**Figure 11:**
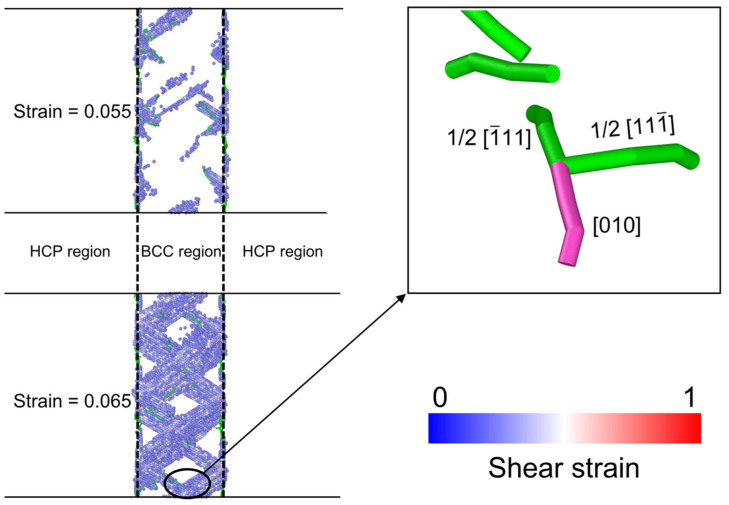
SF distributions in the model with the *T_BCC_* thickness of 10.96 nm at strains of 0.055 and 0.065. The enlarged view in the upper-right corner shows the dislocation structure in the circled region. Atoms are colored by their shear strain values.

At a strain of 0.055, there is a notable increase in the number of S atoms (Figure 10b), which indicates the nucleation of SFs at the phase interface and their propagation into the BCC phase, accompanied by the emission of 1/2<111> dislocations. This deformation mode is similar to the behavior observed in compressed Ti/TiN layered systems [52]. As the strain increases to 0.065, the material experiences a brief softening stage followed by re-hardening. During this stage, intersecting SFs with cross-slip orientations engage dislocation reactions, producing immobile <100> full dislocations. The specific reaction, 1/2[$11\overset{-}{1}$] + 1/2[$\overset{-}{1}$11] = [010], is shown in the enlarged view of Figure 11. From an energetic perspective, the [100] dislocation is lower in energy than two 1/2<111> dislocations and is immobile. Its formation is a crucial microstructural mechanism responsible for the hardening of the material.

**Figure 12:**
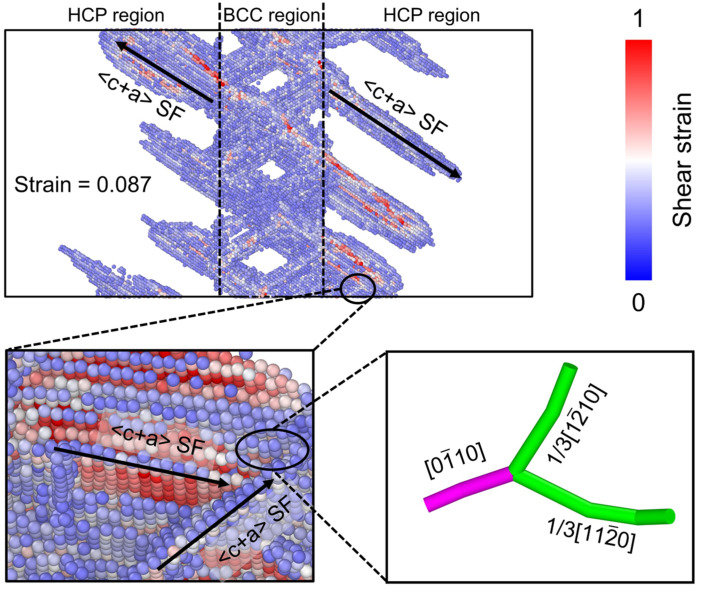
SF distribution of the model with the *T_BCC_* thickness of 10.96 nm at a strain of 0.087. The enlarged view in the lower-left corner shows the SF structure in the circled region, while the one in the lower-right corner shows the corresponding dislocation structure. Atoms are colored by their shear strain values, and the arrows indicate the slip directions of the <c + a> SF.

As deformation progresses toward the peak stress, SFs nucleate at the phase boundary and propagate into the HCP phase (upper panel of Figure 12). At a strain of 0.087, two intersecting <c + a> SFs form a dislocation node structure composed of two 1/3<$1\overset{-}{2}$10> dislocations and one <$1\overset{-}{1}00$> dislocation (lower-left panel of Figure 12). Although these dislocation nodes are locally stable, ongoing SF movement and dislocation reactions lead to significant dislocation multiplication. This process ultimately triggers plastic instability, leading to a drop in stress following the peak. This sequence of microstructural evolution indicates that the change in macroscopic mechanical response originates from plastic instability triggered by SF and dislocations.

**Figure 13:**
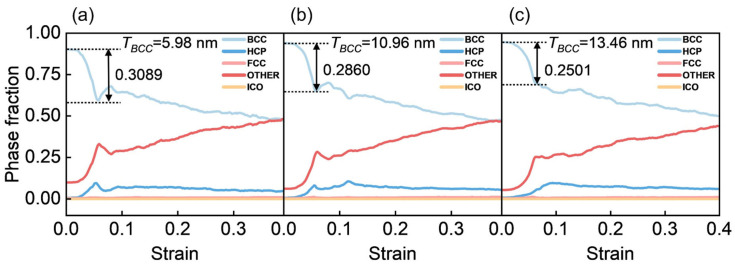
The phase fraction evolution curves of the BCC layer during deformation for (**a**) *T_BCC_* = 5.98 nm, (**b**) *T_BCC_* = 10.96 nm, and (**c**) *T_BCC_* = 13.46 nm models.

**Figure 14:**
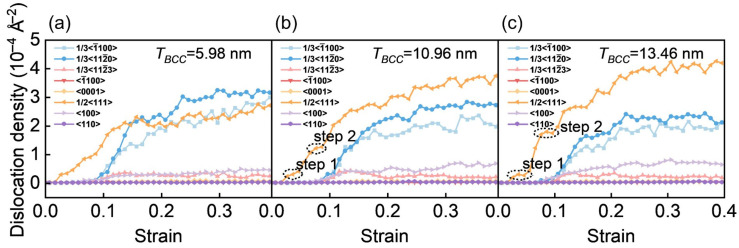
The dislocation density curves for different dislocation types during deformation in models with (**a**) *T_BCC_* = 5.98 nm, (**b**) *T_BCC_* = 10.96 nm, and (**c**) *T_BCC_* = 13.46 nm.

The strain-dependent evolution of BCC phase fraction for the three models is shown in Figure 13a–c. Quantitative analysis indicates that the degree of phase transformation decreases with increasing *T_BCC_*, demonstrating that a thicker BCC layer suppresses phase transformation. This suppression of phase transformation leads to enhanced softening at the second yield point for intermediate *T_BCC_* values, explaining why the *T_BCC_* = 10.96 nm model exhibits pronounced softening behavior, whereas the *T_BCC_* = 5.98 nm model does not (Figure 10a). This softening phenomenon, caused by reduced phase transformation, aligns with previous findings in high-entropy alloys and titanium alloys [58,59]. When *T_BCC_* increases to 13.46 nm, the second yield point starts to increase instead of decrease, indicating that phase transformation-induced strengthening is no longer the dominant mechanism once *T_BCC_* exceeds the critical threshold of 10.96 nm. At this point, a different strengthening mechanism takes precedence. As shown in Figure 11 and Figure 14, interlayer interactions and dislocation reactions significantly enhance the material’s hardening capacity. The development of these microstructures requires sufficient interlayer spacing. This strengthening mechanism is herein referred to as the spatial effects, where increased interlayer spacing facilitates SF propagation, dislocation interactions, and microstructural evolution, ultimately leading to enhanced mechanical performance.

Figure 14a–c present the strain-dependent evolution of dislocation density for various dislocation types in the three models. A comparison with the stress–strain response (Figure 10a) indicates that the emergence of the second yield point is closely associated with the suppression of 1/2<111> dislocation propagation in the BCC phase. The circled regions in Figure 14 (Step 1 and Step 2) exhibit two distinct step-like features in the dislocation density curve, reflecting a stage-wise deceleration in the accumulation of 1/2<111> dislocations. This behavior becomes more pronounced with increasing BCC layer thickness. To understand the physical origin of the step-like features in the dislocation density curves, the *T_BCC_* = 10.96 nm model (Figure 14b) is taken as the reference case. In this model, the two step-like segments in the evolution of 1/2<111> dislocation density can be directly correlated with the defect configurations shown in Figure 11 and Figure 12.

The left endpoint of the first step (Step 1) corresponds to the onset of the second yielding and marks the beginning of softening, consistent with the BCC SF slip observed at a strain of 0.055 (Figure 11). The right endpoint of Step 1 represents dislocation recovery and multiplication in the BCC phase, in agreement with the formation of new dislocations through SF intersections at a strain of 0.065 (Figure 11), leading to re-hardening. The left endpoint of the second step (Step 2) corresponds to the initiation of SF slip in the HCP phase prior to the peak stress, indicating a transfer of dominant deformation activity from the BCC phase to the HCP phase. The right endpoint of Step 2 marks the onset of intensive dislocation multiplication triggered by SF intersections in the HCP phase (Figure 12).

In general, more pronounced step-like features indicate a stronger strengthening influence of spatial effects. As *T_BCC_* increases from 5.98 nm to 13.46 nm (Figure 14a–c), the step-like characteristics in the dislocation density curves become increasing evidently, suggesting an enhanced stage-wise regulation of dislocation evolution. When *T_BCC_* reaches 13.46 nm, the spatial effects become dominant. Based on the reference interpretation established for *T_BCC_* = 10.96 nm, the more pronounced step-like features observed at 13.46 nm represent an intensified manifestation of the same sequential deformation processes, namely the suppression and subsequent reactivations of 1/2<111> dislocations. The enlarged slip space of the SF in the BCC phase allows for the sustained accumulation of 1/2<111> dislocations between Step 1 and Step 2, thereby enhancing plastic deformability. Meanwhile, intensified dislocation multiplication effectively alleviates local stress concentrations in the BCC phase, resulting in a reduced hardening slope during the re-hardening stage that follows the second yield point (Figure 10a). In addition, sustained long-range SF slip delays the onset of peak stress. Collectively, the strengthened step characteristics at larger *T_BCC_* values originate from the amplified spatial effects, which reinforce stage-wise dislocation modulation and ultimately improve plastic deformation performance.

Overall, the step-like features observed in the dislocation density curves indicate that spatial effects contribute to strengthening. These effects arise from enhanced interactions between SFs and dislocations under favorable geometrical conditions and are closely coupled with interfacial-mediated deformation compatibility. The relationship between peak stress and *T_BCC_* is influenced by the competition between transformation-induced hardening in the metastable BCC phase and dislocation-mediated strengthening associated with spatial effects. Previous MD studies have shown that BCC → HCP phase transformation is often associated with an increase in stress during deformation. This has been observed in materials such as Cu thin films, high-entropy alloys, and FCC/BCC multilayers [25,60,61]. In the metastable β (BCC) Ti–Mo system, the transformation of the β phase also contributes to enhanced strength during tensile testing [59]. However, in Zr–Nb alloys, such transformation-induced strengthening has not been explicitly established. Our simulations provide clear evidence of transformation-induced strengthening from the BCC phase in Zr–Nb system. Additionally, we found that this strengthening effect diminishes as the thickness of the BCC layer increases.

When *T_BCC_* is smaller than 10.96 nm, transformation-induced hardening dominates the mechanical response. In this regime, the BCC → HCP transformation provides a major contribution to strengthening, while dislocation activity remains relatively limited due to spatial confinement. As *T_BCC_* increases, the phase transformation is progressively suppressed, leading to a reduction in transformation-induced hardening and a corresponding decrease in peak stress. At the same time, the increased layer thickness promotes dislocation activity within the BCC phase.

When *T_BCC_* exceeds 10.96 nm, dislocation-mediated strengthening becomes the dominant mechanism. Enhanced dislocation interactions, such as cross-slip and dislocation pile-up, intensify strain hardening. This observation aligns with experimental findings in AlCoCrFeNi_2.1_ (FCC/BCC) multilayer systems, where an increase in layer spacing shifts the dislocation behavior from single slip to cross-slip and pile-up at the interfaces, which contributes to strengthening [16]. Meanwhile, the larger spatial volume provides enough room for defect nucleation and propagation, thereby improving plastic deformability.

It is worth noting that dislocation pile-up at the HCP/BCC phase boundary has been experimentally observed in Zr–2.5Nb alloys [11]. The accumulation of these piled-up dislocations generates a larger back stress, which contributes to strain hardening during deformation [10]. Our results provide further insights into how dislocation activity evolves within the BCC phase—an aspect that is challenging to investigate experimentally. The simulations show that as the BCC phase thickness increases, both the cross-slip of dislocations within the phase and the strengthening effect due to dislocation pile-up at the phase boundary become more pronounced. In addition, experiments on Zr/Nb layered materials have shown that when the period thickness is below 27 nm, the hardness decreases; the thinner the HCP (Zr) layer, the softer the material. This phenomenon is essentially attributed to the enhanced ability of slip bands to cross the phase boundary and propagate across layers [14]. In this study, the strengthening effect, as indicated by the peak stress within the BCC layer thickness range of 10.96 nm to 13.46 nm, aligns well with the experimental trend. This explains why the barrier effect of the phase boundary against dislocations originating from the BCC phase becomes stronger as *T_BCC_* increases.

Consequently, at *T_BCC_* = 13.46 nm, an optimal balance between transformation-induced and dislocation-mediated strengthening is achieved, leading to a pronounced strength–ductility synergy. Additionally, regarding the influence of factors such as strain rate and temperature on critical thickness, previous studies on other material systems (such as Cu/Ag multilayers [62], Ni-based alloys [63], and Mg [64]) have found that these effects are not significant. Whether this perspective can be extended to the Zr–Nb alloy systems will be a direction for future research.

## 4. Conclusions

Molecular dynamics simulations demonstrate that the mechanical response and the strength–ductility synergy in nanolayered Zr–Nb dual-phase alloys are governed by the coupling between interfacial strain coordination and BCC layer thickness-dependent deformation competition. The following conclusions can be drawn:

1. The strain coordination at the interface determines the deformation mode. When the loading orientation promotes compatibility of strain across the HCP/BCC interface, the sustained plastic behavior of the metastable BCC phase facilitates SF nucleation, dislocation transmission, and activation of <c + a> slip in the HCP phase. This cooperative deformation enhances the capacity for strain hardening and delays instability. In contrast, strain incompatibility suppresses BCC plasticity and limits high-CRSS slip activation.
2. The thickness of BCC layer induces a non-monotonic peak stress transition influenced by competition mechanisms. For *T_BCC_* < 10.96 nm, the stress-induced β-phase transformation is the dominant mechanism for strengthening. Increasing *T_BCC_* suppresses this transformation activity, leading to a reduction in peak strength. When *T_BCC_* exceeds the transition thickness (~10.96 nm), spatial effects become dominant, causing a shift in the governing mechanism from transformation-induced hardening to dislocation-mediated strengthening.
3. The stage-wise dislocation evolution stabilizes plastic flow at large *T_BCC_* values. Thick BCC layers exhibit step-like increments in dislocation density due to intensified interactions among 1/2<111> dislocations and enhanced SF propagation. This dynamic balance between dislocation multiplication and annihilation stabilizes strain hardening and ultimately influences the delayed peak stress behavior.

## Data Availability

The original contributions presented in this study are included in the article/Supplementary Material. Further inquiries can be directed to the corresponding author.

## Supplementary Materials

The following supporting information can be downloaded at: https://www.mdpi.com/article/10.3390/ma19071398/s1, Figure S1: Comparison of peak compressive stress and flow stress from MC/MD simulations using 200,000 (C1) and 400,000 (C2) cycles for HCP/BCC models with *T_BCC_* = 3.99–13.46 nm. Both sets show identical stress trends across all *T_BCC_* values, confirming the robustness of the results and their insensitivity to stochastic effect; Figure S2: Evolution of (a) potential energy, (b) total energy, (c) temperature, and (d) average atomic volume during the cooling–relaxation process.

## References

[1] Song G., Zhang C., Xin Y., Huang X., Wu P., Zhou J., Zhu W., Zhou X. (2025). The mechanism for the self-accommodation microstructure of α variants during phase transformation of the Zr–2.5Nb alloy. J. Mater. Sci. Technol..

[2] Li J., Cui Y., Wu H., Chen G. (2020). Deformation mechanism of Zr–Sn–Nb–Fe cladding tube under various stress states. Mater. Sci. Eng. A.

[3] Wang S., Giuliani F., Ben Britton T. (2020). Slip–hydride interactions in Zircaloy-4: Multiscale mechanical testing and characterisation. Acta Mater..

[4] Holt R.A. (2008). In-reactor deformation of cold-worked Zr-2.5Nb pressure tubes. J. Nucl. Mater..

[5] Griffiths M., Winegar J.E., Buyers A. (2008). The transformation behaviour of the β-phase in Zr-2.5Nb pressure tubes. J. Nucl. Mater..

[6] Kulkarni R.V., Krishna K.V.M., Neogy S., Srivastava D., Ramadasan E., Shriwastaw R.S., Rath B.N., Saibaba N., Jha S.K., Dey G.K. (2014). Mechanical properties of Zr-2.5%Nb pressure tube material subjected to heat treatments in α plus β phase field. J. Nucl. Mater..

[7] Zhilyaev A.P., Sabirov I., Gonzalez-Doncel G., Molina-Aldareguia J., Srinivasarao B., Perez-Prado M.T. (2011). Effect of Nb additions on the microstructure, thermal stability and mechanical behavior of high pressure Zr phases under ambient conditions. Mater. Sci. Eng. A.

[8] Yang Z.N., Wang X.B., Liu F., Zhang F.C., Chai L.J., Qiu R.S., Chen L.Y. (2019). Effect of intercritical annealing temperature on microstructure and mechanical properties of duplex Zr-2.5Nb alloy. J. Alloys Compd..

[9] Muránsky O., Daymond M.R., Bhattacharyya D., Zanellato O., Vogel S.C., Edwards L. (2013). Load partitioning and evidence of deformation twinning in dual-phase fine-grained Zr–2.5%Nb alloy. Mater. Sci. Eng. A.

[10] Zhang J.W., Beyerlein I.J., Han W.Z. (2019). Hierarchical 3D Nanolayered Duplex-Phase Zr with High Strength, Strain Hardening, and Ductility. Phys. Rev. Lett..

[11] Zou X.W., Beyerlein I.J., Han W.Z. (2024). Hierarchical nanolayered structures-enabled record-high fracture resistant zircaloy. Acta Mater..

[12] Jiang S., Peng R.L., Máthis K., Yan H.-L., Farkas G., Hegedues Z., Lienert U., Moverare J., Zhao X., Zuo L. (2022). Shear banding-induced <c+a> slip enables unprecedented strength-ductility combination of laminated metallic composites. J. Mater. Sci. Technol..

[13] Long F., Balogh L., Brown D.W., Mosbrucker P., Skippon T., Judge C.D., Daymond M.R. (2016). Effect of neutron irradiation on deformation mechanisms operating during tensile testing of Zr-2.5Nb. Acta Mater..

[14] Callisti M., Polcar T. (2017). Combined size and texture-dependent deformation and strengthening mechanisms in Zr/Nb nano-multilayers. Acta Mater..

[15] Ham B., Zhang X. (2011). High strength Mg/Nb nanolayer composites. Mater. Sci. Eng. A.

[16] Ji W., Gao S., Jarlöv A., Shen X., Tian Y., Wu M.S., Gao H., Zhou K. (2025). Designing Maximal Strength in Nanolamellar Eutectic High-Entropy Alloys. Adv. Mater..

[17] Cai S., Daymond M.R., Holt R.A., Gharghouri M.A., Oliver E.C. (2009). Evolution of interphase and intergranular stresses in Zr-2.5Nb during room temperature deformation. Mater. Sci. Eng. A.

[18] Li W., Yu W., Xu Q., Zhou J., Nan H., Yin Y., Feng X., Shen X. (2020). Effects of γ/γ interfaces in TiAl lamellae subjected to uniaxial tensile loading. Comput. Mater. Sci..

[19] Li W., Yin Y., Xu Q., Zhou J., Nan H., Ji X., Shen X., Feng X., Yu W., Tu Z. (2019). Tensile behavior of γ/α2 interface system in lamellar TiAl alloy via molecular dynamics. Comput. Mater. Sci..

[20] Ma G.C., Fan J.L., Gong H.R. (2018). Mechanical behavior of Cu-W interface systems upon tensile loading from molecular dynamics simulations. Comput. Mater. Sci..

[21] Kong X.F., Beyerlein I.J., Liu Z.R., Yao B.N., Legut D., Germann T.C., Zhang R.F. (2019). Stronger and more failure-resistant with three-dimensional serrated bimetal interfaces. Acta Mater..

[22] Yadav S.K., Shao S., Chen Y., Wang J., Liu X.Y. (2017). Atomistic modeling of Mg/Nb interfaces: Shear strength and interaction with lattice glide dislocations. J. Mater. Sci..

[23] Lin B., Li J., Wang Z., Wang J. (2020). Dislocation nucleation from Zr-Nb bimetal interfaces cooperating with the dynamic evolution of interfacial dislocations. Int. J. Plast..

[24] AlMotasem A.T., Daghbouj N., Sen H.S., Mirzaei S., Callisti M., Polcar T. (2023). Influence of HCP/BCC interface orientation on the tribological behavior of Zr/Nb multilayer during nanoscratch: A combined experimental and atomistic study. Acta Mater..

[25] Ju S.P., Huang P.X., Chen H.L., Chen H.T., Chen H.Y., Wu D.Y. (2024). Tailoring strength and ductility in dual-phase high-entropy alloys: Insights from deep learning molecular dynamics simulation on FCC/BCC thickness ratios. J. Mater. Res. Technol..

[26] Plimpton S. (1995). Fast parallel algorithms for short-range molecular dynamics. J. Comput. Phys..

[27] Starikov S., Smirnova D. (2021). Optimized interatomic potential for atomistic simulation of Zr-Nb alloy. Comput. Mater. Sci..

[28] Starikov S., Abbass A., Drautz R., Mrovec M. (2023). Disordering complexion transition of grain boundaries in bcc metals: Insights from atomistic simulations. Acta Mater..

[29] Lin J., Chen S., Bai Y., Zhang S., Wang T., Zhao J. (2024). Atomistic simulations of the interaction of edge dislocations with β-Nb precipitates in Zr-Nb alloys. J. Phys. D Appl. Phys..

[30] Hasan M.M., Srinivasan S.G., Choudhuri D. (2024). Transformation- and twinning-induced plasticity in phase-separated bcc Nb-Zr alloys: An atomistic study. J. Mater. Sci..

[31] Chen Z., Zeng Z., Li H., Song S., Xiang H., Peng X. (2025). Microstructure evolution and spallation of CoCrFeNi/Al multilayers subjected to shock loading. Phys. B.

[32] Zhang J., Qian L., Yang W., Wang J., Yang X.-S. (2025). Dislocation nucleation and shear sliding at dual-phase high-entropy alloy semi-coherent interface with atomic complexity. Acta Mater..

[33] Niu Y., Zhao D., Zhu B., Wang S., Zhang Z., Zhao H. (2024). Research on the effects of chemical short-range order on strengthening and toughening mechanisms of FCC/BCC dual-phase high-entropy alloys at micro/nano-scale. Mater. Today Commun..

[34] Abdolrahim N., Zbib H.M., Bahr D.F. (2014). Multiscale modeling and simulation of deformation in nanoscale metallic multilayer systems. Int. J. Plast..

[35] Han R.Q., Song H.Y., Wang J.Y., Li Y.L. (2021). Strengthening mechanism of Al matrix composites reinforced by nickel-coated graphene: Insights from molecular dynamics simulation. Phys. B Condens. Matter.

[36] Su M.J., Deng Q., An M.R., Liu L.T., Ma C.B. (2019). Molecular dynamics study of the tensile behaviors of Ti(0 0 0 1)/Ni(1 1 1) multilayered nanowires. Comput. Mater. Sci..

[37] Sha Z.-D., Branicio P.S., Lee H.P., Tay T.E. (2017). Strong and ductile nanolaminate composites combining metallic glasses and nanoglasses. Int. J. Plast..

[38] Tran A.-S., Fang T.-H. (2020). Size effect and interfacial strength in nanolaminated Cu/CuxTa100-x composites using molecular dynamics. Comput. Mater. Sci..

[39] Chen Y., Shao S., Liu X.Y., Yadav S.K., Li N., Mara N., Wang J. (2017). Misfit dislocation patterns of Mg-Nb interfaces. Acta Mater..

[40] Cai S., Daymond M.R., Holt R.A. (2012). Deformation of high β-phase fraction Zr-Nb alloys at room temperature. Acta Mater..

[41] Huang Z., Li T., Fang Y., Smith J., Li B., Brozena A., Dong Q., Zhang Q., Du Y., Mao S.X. (2025). Phase Changes of Multielemental Alloy Nanoparticles at Elevated Temperatures. ACS Nano.

[42] Zhao Y., Li H., Huang Y. (2021). The structure, mechanical, electronic and thermodynamic properties of bcc Zr-Nb alloy: A first principles study. J. Alloys Compd..

[43] Antillon E., Woodward C., Rao S.I., Akdim B. (2021). Chemical short range order strengthening in BCC complex concentrated alloys. Acta Mater..

[44] Metropolis N., Rosenbluth A.W., Rosenbluth M.N., Teller A.H., Teller E. (1953). Equation of State Calculations by Fast Computing Machines. J. Chem. Phys..

[45] Stukowski A. (2010). Visualization and analysis of atomistic simulation data with OVITO-the Open Visualization Tool. Modell. Simul. Mater. Sci. Eng..

[46] Tsuzuki H., Branicio P.S., Rino J.P. (2007). Structural characterization of deformed crystals by analysis of common atomic neighborhood. Comput. Phys. Commun..

[47] Stukowski A., Albe K. (2010). Extracting dislocations and non-dislocation crystal defects from atomistic simulation data. Modell. Simul. Mater. Sci. Eng..

[48] Shimizu F., Ogata S., Li J. (2007). Theory of shear banding in metallic glasses and molecular dynamics calculations. Mater. Trans..

[49] Sansoz F., Ke X. (2022). Hall–Petch strengthening limit through partially active segregation in nanocrystalline Ag-Cu alloys. Acta Mater..

[50] Vu T.N., Pham V.T., Fang T.H. (2022). Influences of grain size, temperature, and strain rate on mechanical properties of Al_0.3_CoCrFeNi high-entropy alloys. Mater. Sci. Eng. A.

[51] Zhang L., Lu C., Tieu K. (2016). A review on atomistic simulation of grain boundary behaviors in face-centered cubic metals. Comput. Mater. Sci..

[52] Yang W., Ayoub G., Salehinia I., Mansoor B., Zbib H. (2017). Multiaxial tension/compression asymmetry of Ti/TiN nano laminates: MD investigation. Acta Mater..

[53] Wan P., Huang Q., Li M., Qu P., Wang P., Zhou H., Wang H. (2024). Orientation effect on α/β phase interface mediated deformation mechanism in titanium alloy. Comput. Mater. Sci..

[54] Wu Z., Curtin W.A. (2016). Mechanism and energetics of <c + a> dislocation cross-slip in hcp metals. Proc. Natl. Acad. Sci. USA.

[55] Gong J., Benjamin Britton T., Cuddihy M.A., Dunne F.P.E., Wilkinson A.J. (2015). <a> Prismatic, <a> basal, and <c+a> slip strengths of commercially pure Zr by micro-cantilever tests. Acta Mater..

[56] Wang L., Xu C., Zhu B., Liu J., Liang N., Liu R., Cao Y., Zhao Y. (2025). Short-range ordering suppresses mechanical annealing in CoCrNi alloy nanopillars. Int. J. Mech. Sci..

[57] Friedman L.H., Chrzan D.C. (1998). Scaling theory of the Hall-Petch relation for multilayers. Phys. Rev. Lett..

[58] Li Z., Pradeep K.G., Deng Y., Raabe D., Tasan C.C. (2016). Metastable high-entropy dual-phase alloys overcome the strength–ductility trade-off. Nature.

[59] Sun F., Zhang J.Y., Marteleur M., Gloriant T., Vermaut P., Laillé D., Castany P., Curfs C., Jacques P.J., Prima F. (2013). Investigation of early stage deformation mechanisms in a metastable β titanium alloy showing combined twinning-induced plasticity and transformation-induced plasticity effects. Acta Mater..

[60] Sun B., Ouyang W., Ren J., Mi L., Guo W. (2019). Fcc→bcc→hcp successive phase transformations in the strained ultrathin copper film: A molecular dynamic simulation study. Mater. Chem. Phys..

[61] Wang Z., Li S., Lian H., Zhao Y., Li Z., Zhai Y., Long H., Wang L., Han X. (2025). Shape memory of bcc structured high-entropy-alloy nanowires during room temperature deformation. Microstructures.

[62] Li P., Zhao C., Jiang Y., Cao F., Xiao P., Song Y., Hong Z., Gou S., Liang S. (2024). The relationship between deformation mechanisms and mechanical properties in nanocrystalline Cu/Ag-bilayer alloy. J. Alloys Compd..

[63] Dong H., Xu T., Ning T., Liu M., Wu D., Ma H., Feng Z., Narayanaswamy B., Su R., Wang T. (2023). Atomic simulations on the deformation mechanisms in nano-crystalline Ni–Al series Ni-based superalloy based on grain size, strain rate and temperature. J. Mater. Res. Technol..

[64] Chandiran E., Ogawa Y., Ueji R., Somekawa H. (2023). An inverse Hall-Petch relationship during room-temperature compression of commercially pure magnesium. J. Alloys Compd..

