# Supplementary material for: Molecular Dynamics Simulation of the Mechanical Properties of Nanolayered Zr-Nb Alloys: Effects of Orientation and Layer Thickness
Source: Materials (Basel). 2026 Mar 31;19(7):1398. doi: 10.3390/ma19071398 (PMC13073849; doi:10.3390/ma19071398)
Supplement: Supplementary file 1 [file materials-19-01398-s001.zip › materials-4215160-supplementary.pdf]

Supplementary material

Molecular dynamics simulation of the mechanical properties of nanolayered Zr-Nb alloys: Effects of orientation and layer thickness

Fugen Deng [1], Guiyu Liu [1], Jianhao Yan [1], Yulu Zhou [1,*] , Yifang Ouyang [1]

[1] School of Physical Science and Technology, State Key Laboratory of Featured Metal Materials and Life-cycle Safety for Composite Structures, Guangxi Key Laboratory for Relativistic Astrophysics, Guangxi University, Nanning 530004, China

* **Correspondence:** ylzhou@gxu.edu.cn

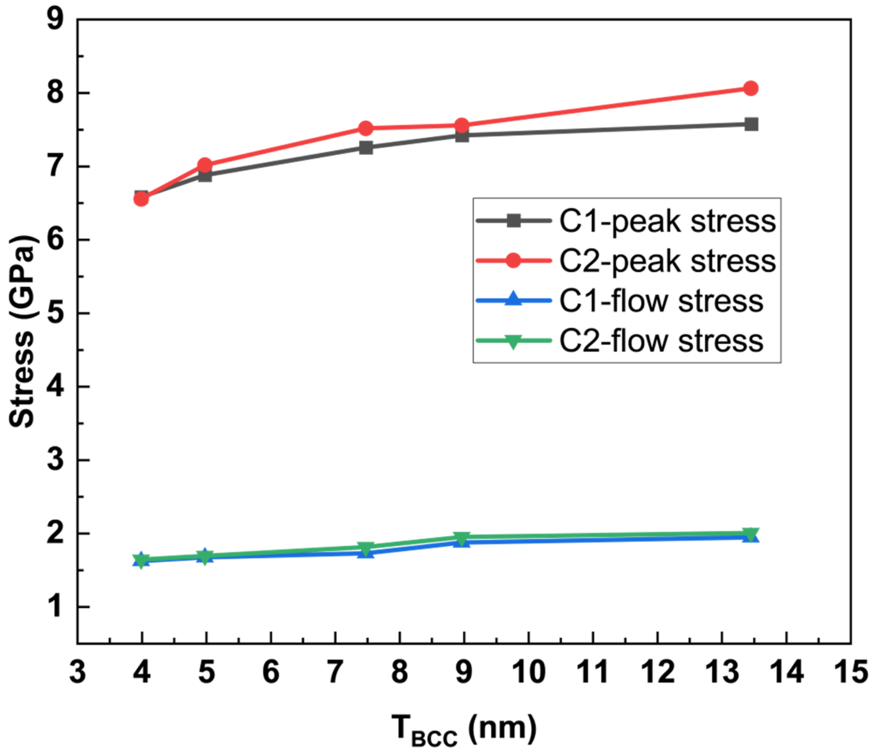

Figure S1. Comparison of peak compressive stress and flow stress from MC/MD simulations using 200,000 (C1) and 400,000 (C2) cycles for HCP/BCC models with T_{BCC} = 3.99–13.46 nm. Both sets show identical stress trends across all T_{BCC} values, confirming the robustness of the results and their insensitivity to stochastic effects.

Based on models with T_{BCC} values of 3.99, 4.98, 5.98, 7.48, 8.97, 10.96, and 13.46 nm, two sets of simulations were performed using different numbers of MC/MD cycles. Models simulated with 200,000 MC/MD cycles were designated as group C1, while those simulated with 400,000 cycles were designated as group C2. Identical compressive loading conditions were applied to both groups of HCP/BCC structures. As shown in Fig. S1, both the peak compressive stress and flow stress exhibit the same dependence on BCC layer thickness in the two groups. This consistency excludes stochastic effects arising from the hybrid MC/MD procedure and confirms the robustness and reliability of the present analysis.

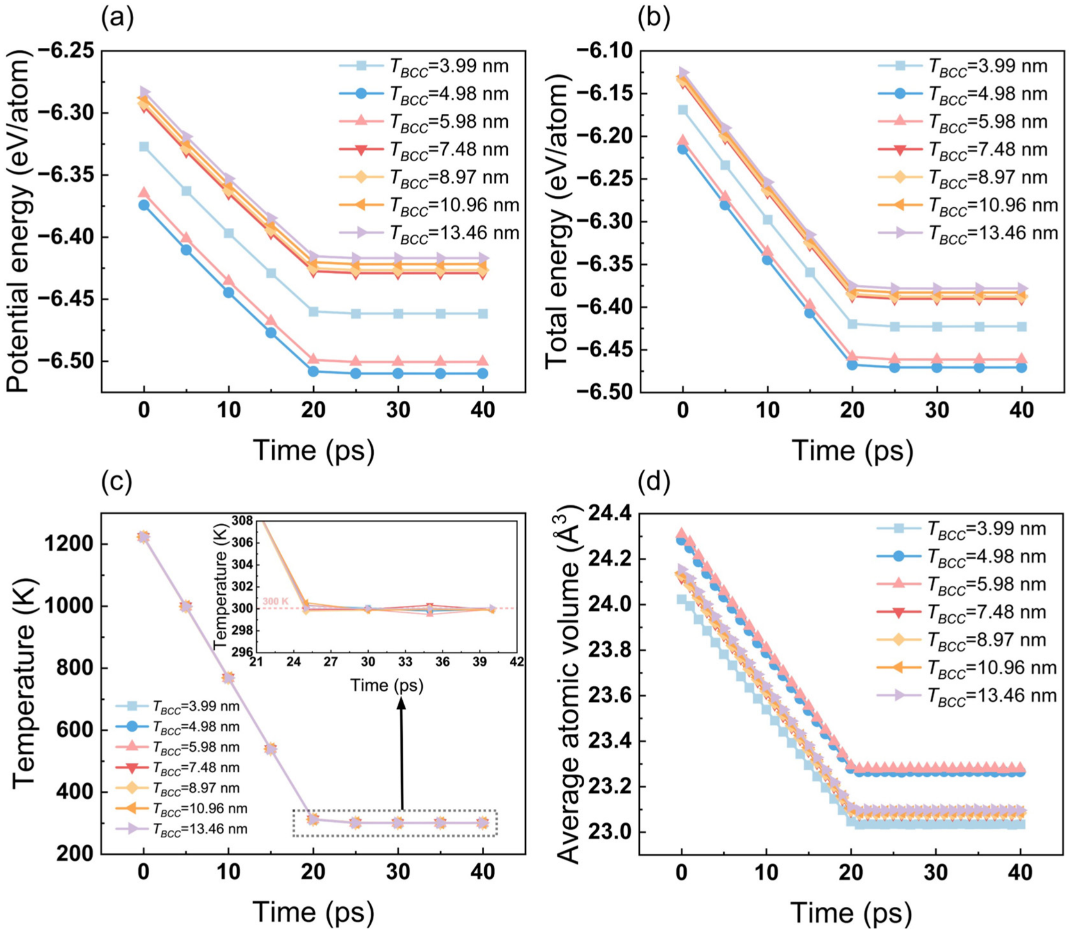

Figure S2. Evolution of (a) potential energy, (b) total energy, (c) temperature, and (d) average atomic volume during the cooling–relaxation process.

Figure S2 shows the temporal evolutions of energy, temperature, and average atomic volume during the cooling stage (0 – 20 ns) and the subsequent relaxation stage (20 – 40 ns) after the MC/MD treatment. During the relaxation stage, the system is maintained at 300 K. Both the energy and atomic volume curves have converged, indicating that the model has reached a thermally stable state.
